# New Nanomaterials with Intrinsic Antioxidant Activity by Surface Functionalization of Niosomes with Natural Phenolic Acids

**DOI:** 10.3390/pharmaceutics13060766

**Published:** 2021-05-21

**Authors:** Elisabetta Mazzotta, Carla Orlando, Rita Muzzalupo

**Affiliations:** 1 Centro di Ricerca Olivicoltura, Frutticoltura, Agrumicoltura, Consiglio per la Ricerca in Agricoltura e L’analisi dell’Economia Agraria (CREA-OFA), 87036 Rende, Italy; mazzotta-elisabetta@libero.it; 2 Department of Pharmacy, Health and Nutritional Sciences, University of Calabria Via Pietro Bucci, Ed. Polifunzionale, 87036 Arcavacata di Rende, Italy; carla.orlando96@gmail.com

**Keywords:** nanoantioxidant carrier, non-ionic surfactant vesicles, antioxidant grafting, DPPH, synergic therapy

## Abstract

Nanoantioxidants have emerged as smart devices able to provide improved stability and biocompatibility and sustained and targeted release of conventional antioxidants. In the current research, a new family of nanoantioxidants has been developed by covalently grafting gallic (GA), caffeic (CF) and ferulic (FR) acid on the surfaces of Tween 80 niosomes. First, empty and curcumin (CUR)-loaded vesicles were prepared using a thin-layer evaporation technique and then functionalized with phenolic acids using carbodiimide chemistry. Nanoantioxidants obtained were characterized in terms of size, polydispersity index, zeta potential, and loading efficiency. Their antioxidant activity was studied by ABTS and DPPH assays. Surface functionalization of empty and CUR-loaded vesicles provided stable vesicles with intrinsic antioxidant properties. In vitro antioxidant assays highlighted that vesicles functionalized with FR or GA exhibited better antioxidant activity compared to CF-grafted niosomes. Furthermore, vesicles loaded with CUR and functionalized with GA and CF showed an enhanced scavenging ability of ABTS and DPPH radicals, compared to the single antioxidant-loaded formulations, highlighting an important synergic effect of CUR when used in combination with GA ad CF.

## 1. Introduction

Antioxidants, recognized as prophylactic and therapeutic molecules, are utilized in a number of applications in the pharmaceutical, cosmetic and nutraceutical fields for the countless healthy benefits associated with their use [1]. The involvement of antioxidants in several redox cellular pathways has been intensively studied and further studies are ongoing from scientists to enhance their role of defense against oxidative stress. Oxidative stress based on the overproduction of reactive oxygen species (ROS) is commonly associated with different pathophysiological processes, such as cancer, atherosclerosis, diabetes, Alzheimer’s disease, and Parkinson’s disease [2,3,4]. Antioxidants play a critical role in disease prevention and treatment, maintaining a healthy redox balance between pro- and antioxidant species, thanks to their ability to delay or inhibit the undesired oxidative degradation of biological macromolecules to free radicals.

Despite their huge potential, the application of antioxidants is often limited due to susceptibility to light, oxygen, and pH, poor solubility in physiological fluid, low bioavailability, and improper delivery in undesired cellular compartments [5,6,7].

In the last decade, nanotechnology has been applied in antioxidant delivery, leading to the development of smart nanocarriers endowed with antioxidant properties, known as ‘nanoantioxidants’ [8]. Nanoantioxidant systems could overcome many limitations of traditional antioxidant molecules and improve their efficacy, thanks to their prolonged stability, improved bioavailability, the ability to avoid fast metabolic clearance, and to provide a controlled and targeted delivery [9].

The free radical scavenging activity of these carriers could be related to the passive delivery of small antioxidant molecules or some intrinsic antioxidant properties of the device. Encapsulation technology is based on the loading of antioxidant molecules in drug delivery systems, such as liposomes [10], niosomes [11], solid lipid nanoparticles [12], and polymeric nanoparticles [13], in order to provide devices able to protect antioxidants and enhance their absorption. On the other hand, some inorganic nanoparticles, such as gold, cerium, and iron nanoparticles, have emerged as devices with intrinsic antioxidant activity related to a radical trapping ability [14].

Another promising strategy involves the surface functionalization of nanocarrier surfaces with small antioxidant molecules to transform inactive devices into antioxidants with improved characteristics. This approach has been actively pursued in recent years and represents an intriguing way not only to provide antioxidant devices, but also to improve several nanocarriers’ properties, such as biocompatibility, biostability, and the ability to escape immune system activation [15]. Specifically, the simultaneous loading and functionalization of nanocarriers with antioxidants provides the advantage of delivering high antioxidant amounts and the possibility for the co-delivery of other drugs and, thus, for the use of these devices to exploit any synergic effects [16]. The functionalization of nanodevices with natural antioxidants also allows them to be conferred their specific biological activity, depending on the molecule used for the functionalization process, such as antidiabetic [17], antimicrobial, anticancer [18], and anti-Alzheimer’s disease [19]. Considering these advantages, Elle et al. [20] proposed the surface functionalization of mesoporous silica nanoparticles with rutin and caffeic acid as a successful strategy to reduce ROS production and generate safer devices for different pharmaceutical applications. In another study, iron magnetic nanoparticles functionalized with gallic acid exhibited synergistic organic–inorganic hybrid antioxidant properties and potent antimicrobial activity on different bacterial and fungal strains [21].

To the best of our knowledge, no studies focusing on niosomes with intrinsic antioxidant properties have been reported in the literature.

In recent years, niosomes, versatile nanosized devices, have been pursued as an increasing research interest for their excellent properties, such as high surface area, high stability, and wide versatility in the composition due to the large availability of non-ionic surfactants [22]. Specifically, the large surface of niosomal vesicles makes them suitable for chemical modifications and offers the possibility to easily customize them to a particular and desired therapeutic application.

Herein, we aimed to synthesize niosomes with intrinsic antioxidant properties by chemical conjugation of phenolic acids onto vesicle surfaces.

Phenolic compounds are secondary plant metabolites and represent the most common molecules with antioxidant activity present in dietary sources, such as vegetables, grapes, red wine, fruit juices, tea, and coffee. These micronutrients, in addition to their potent antioxidant activity, are known to exert a variety of pharmacological effects, such as anti-inflammatory, antimicrobial, and anticancer activities [23,24]. It is in our interest, thus, to conjugate these molecules on niosome surfaces, since this could be an efficient strategy to personalize these carriers for a specific therapeutic application and to use these devices as carriers of another drug for a potential combination therapy.

Due to their important antioxidant activity and different biological properties, gallic (GA), ferulic (FR), and caffeic acid (CF) were chosen as antioxidant components of the new nanomaterials. We set out to investigate whether functionalization of these polyphenols onto Tween 80 niosomes’ surfaces could provide them with antioxidant properties. After functionalization through chemistry carbodiimide, the developed vesicles were characterized, and their antioxidant activity was evaluated in vitro using 2,2-diphenyl-1-picrylhydrazyl (DPPH) and 2,2-azinobis-(3-ethylbenzothiazoline-6-sulfonate) (ABTS) assays to investigate the potential of these devices as new antioxidant systems. Furthermore, we also set out to use these systems as carriers of other drugs for a potential synergic therapy. For this purpose, curcumin (CUR), a natural polyphenol widely employed to reduce oxidative stress in many pathologies [25], was loaded into the vesicles’ bilayer with the aim of evaluating the potential synergic effects and increased biological activity.

## 2. Materials and Methods

### 2.1. Chemicals

Tween 80, curcumin, gallic acid, ferulic acid, caffeic acid, 2,2-diphenyl-1-picrylhydrazyl (DPPH), 2,2-azinobis-(3-ethylbenzothiazoline-6-sulfonate) (ABTS), and sepharose CL-4B gel were purchased from Sigma–Aldrich (Milan, Italy). All solvents for the high-performance liquid chromatography were used and provided by VWR International Srl (Milan, Italy). All chemicals were used without further purification.

### 2.2. Preparation of Niosomes

Tween 80 multilamellar niosome vesicles (MLVs) were prepared by the traditional thin-film hydration method. In order to obtain CUR-loaded niosomes, 2.7 × 10^−6^ moles of CUR were added to the organic surfactant solution. Small unilamellar vesicles (SUVs) were obtained from MLVs by sonication. The purification method of formulation was gel permeation chromatography. The samples were stored in the dark at 4 °C until their use in subsequent experiments. CUR entrapment efficiency was then determined using a UV–vis spectrophotometer at 426 nm and was reported as the percentage of drug loaded into niosomes versus the initial total drug.

Hydrodynamic diameter of niosomes and zeta potential were determined by dynamic/electrophoretic light scattering at 25 ± 0.1 °C. More details are provided in the Supplementary Materials.

### 2.3. Preparation of GA-, CF-, FR-, Conjugated Niosomes

**Figure 1:**
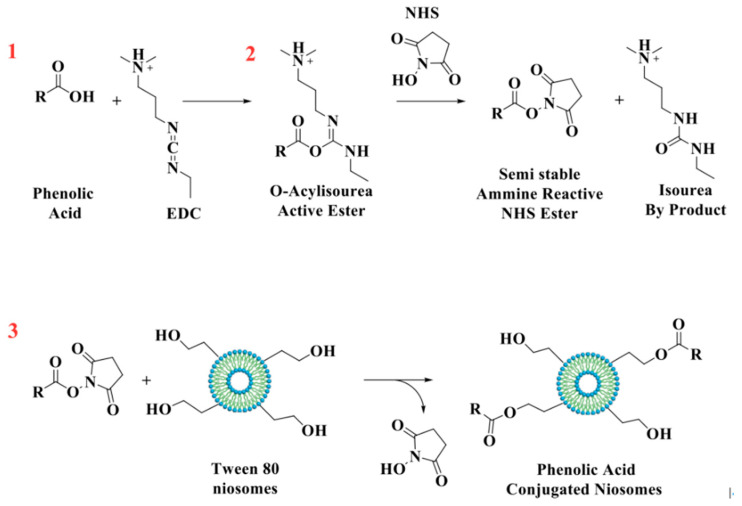
Schematic representation of the reaction between antioxidant molecules and niosomes.

GA, CF, and FR were chemically grafted onto niosomes’ surfaces using EDC/NHS coupling agents (Figure 1) [26]. Briefly, phenolic compounds (0.01 mmol), EDC (0.01 mmol), and NHS (0.01 mmol) were dissolved in ethanol and stirred in an ice bath for 1 h. In the next steps, 0.04 mmol of surfactant mixture was added and incubated overnight at room temperature. Free phenolic compounds and other byproducts (isourea) were removed by dialysis for 4 h.

### 2.4. Determination of Total Phenolic Content

Total phenolic content present in the formulations was evaluated by the Folin–Ciocalteu method [27]. A calibration curve using GA was carried out and the total phenolic content was expressed as GA equivalents (GAE). Grafting efficiency (%) was determined by measuring the amount of phenolic acids conjugated on the vesicles’ surfaces after purification for dialysis, according to the following equation:

(1) $$\mathrm{Grafting}\;\mathrm{efficiency}\left(\%\right)=\frac{\mathrm{phenol}\;\mathrm{content}\;\mathrm{after}\;\mathrm{dialysis}}{\mathrm{phenol}\;\mathrm{content}\;\mathrm{before}\;\mathrm{dialysis}}\times 100$$

### 2.5. DPPH and ABTS Radical Scavenging Activity Assay

The antioxidant activity was determined by using a DPPH assay according to the method reported by Tavano et al. [11], while the ABTS assay was performed by measuring their capacity to scavenge free radical ABTS [28]. More details are provided in the Supplementary Materials.

### 2.6. In Vitro CUR Release Studies

The CUR release (2.84 × 10^−8^ moles) from antioxidant vesicles was examined under sink conditions in dialysis bags, suspended in 20 mL of phosphate buffer, pH 7.4, containing 0.5% of Tween 80, under stirring for 24 h at 37 °C. At specific time points, 2 mL of the medium was taken and withdrawn with 2 mL of the fresh buffer. The amount of CUR in the withdrawn samples was analyzed using a UV–vis spectrometer at 426 nm and determined according to a calibration curve, prepared for the curcumin solutions of known concentrations in the appropriate range [29].

### 2.7. Statistical Analysis

All data were expressed as the mean ± SD of three independent experiments. Statistical significance was calculated by one-way analysis of variance (ANOVA) and Bonferroni-corrected *p*-value for multiple comparison test. The level of statistically significant difference was defined as *p* < 0.05.

## 3. Results

### 3.1. Niosomes’ Characterization

In recent years, the design of tailor-made carriers has been actively pursued as a promising way to achieve a personalized therapy. Specifically, functionalization of a nanocarrier surface is an interesting approach used to develop smart devices with specific properties dependent on the molecule used for the grafting process. Niosomes are one of the most used candidates to develop smart nanocarriers for pharmaceutical applications. Indeed, the large availability of functional groups on the hydrophilic surfaces of vesicles allows for easy functionalization.

Herein, novel antioxidant nanodevices were designed through surface functionalization of traditional niosomes with natural polyphenols. Specifically, we selected a Tween 80 surfactant which is rich in –OH terminal groups. Vesicles were prepared and then functionalized using EDC as a coupling agent in order to enhance the reaction of the carboxylic group of phenolic acids with –OH groups on the niosomes’ surfaces.

**Figure 2:**
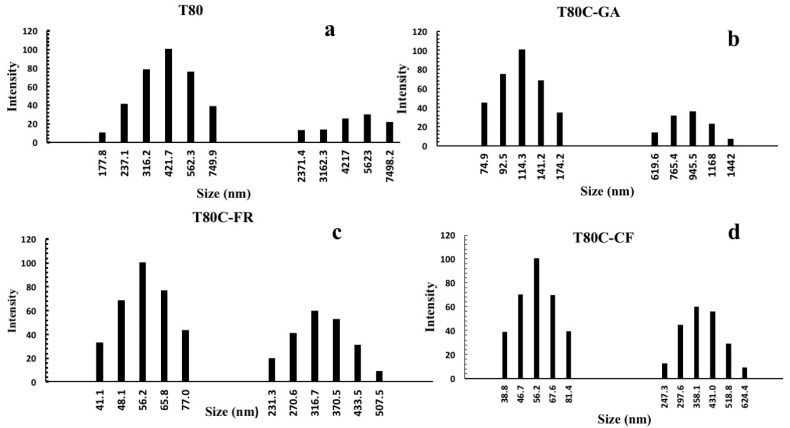
Size distribution of niosomes before (**a**) and after CUR loading and functionalization with GA (**b**), FR (**c**), and CF (**d**).

A Tween 80 surfactant resulted in forming stable vesicles of uniform size of around 549.8 nm and these results are similar to a previous study [30]. Niosomes’ sizes were strongly affected by drug loading and surface functionalization, as shown in Figure 2. The conjugation of phenolic acids on the niosomes’ surfaces decreased the diameter of niosomes by up to 495.8, 433.8, 373.1 nm after conjugation with GA, CF, and FR, respectively (Table 1).

This could be explained by the hydrophobic nature of phenolic acids and specific interaction occurring between them and the surfactant. Several studies, in fact, showed the formation of intermolecular hydrogen bonds between phenolic compounds and a lipid bilayer as the main cause of the higher membrane cohesion and, subsequently, the reduced vesicle size [31,32]. The phenolic acid/surfactant interactions increased the hydrophobic attraction forces developed among surfactant head-groups, resulting in a smaller surface area for molecules and in a dense and compact structure [33,34]. Another reason could be related to the hydrophobic character of the molecule conjugated on the vesicles’ surfaces. In fact, the vesicle size became dependent on the hydrophilic–lipophilic balance of the formulation and the increase in hydrophobicity is commonly associated with the decreased surface-free energy and, consequently, reduced vesicle sizes [35,36]. Furthermore, the decrease in niosome size became dependent on the lipophilicity of phenolic acids conjugated on the vesicles’ surfaces: the higher the lipophilicity, the smaller the sizes that were obtained. In fact, the log P of GA is 0.7, indicating a lower lipophilic character in respect to FR and CF, which present a log P equal to 1.67 and 1.53, respectively. Consequently, GA functionalization led to a coating layer on the vesicle with lower cohesion and, consequently, higher sizes in respect to that obtained with FR and CF.

With the aim of evaluating any synergic effect, we loaded in a vesicle bilayer CUR, a natural polyphenol with several reported possibilities in the treatment of different diseases, such as cancer, inflammation, arthritis, and metabolic disorders [37]. The pharmacological effects of this compound are associated with its strong antioxidant activity and different studies have indicated an important antioxidant synergic effect when used in combination with GA [38], resveratrol [39], and quercetin [40].

In light of our results, we decided to investigate whether the combination of CUR and the new antioxidant niosomes could provide similar results.

Relevantly, the loading of the drug affected the vesicle sizes (Table 2) (Figure 2). Due to its lipophilic nature, CUR is completely embedded in the lipid bilayer and forms hydrogen bonds with surfactant molecules that results in a decrease in the vesicle sizes. In the literature, the similarity of CUR to cholesterol as a condensing agent on the vesicle membrane has been widely reported [41,42]. Cholesterol results in increased membrane packing, leading to a reduced size of niosomal formulations [43,44]. In any case, this smaller size is a desirable feature for drug delivery systems since it is a crucial factor influencing several parameters, such as better stability in vivo, extended bioavailability, reduced selective uptake by the reticuloendothelial system (RES), and the ability to deliver drugs to targets that are difficult to reach [45,46].

The polydispersity index of the samples ranged from 0.187 to 0.281, indicating the good homogeneity and quality of niosomal formulations. All developed nanosystems were, in the long term, stable, and any creaming, sedimentation, and flocculation was observed after 3 months of storage in the dark at room temperature.

CUR was successfully entrapped in the niosomal bilayer: in particular, 7.1 × 10^−7^ moles of the drug, corresponding to approximately 26.30%, were loaded and any drug leakage was observed during the functionalization process.

The Folin–Ciocalteu method was used to determine the total amount of phenolic acids grafted onto the niosomal surface using GA as a standard. The amount of polyphenol grafted on the niosomal surface was 0.11, 0.19, and 0.15 mg GAE/mL for T80-GA, T80-CF, and T80-FR, respectively.

Grafting efficiency was further assessed using UV–vis spectroscopy and the data obtained (Table 1) confirmed the results obtained with the Folin–Ciocalteu method.

### 3.2. Antioxidant Activity

In order to investigate whether the chemical conjugation of phenolic acids on the niosomal surface provided them with antioxidant activity, DPPH and ABTS assays were performed.

A DPPH test is an easy and fast method that involves the reduction of DPPH• radicals in the non-radical form, resulting in a change in the color of the solution from purple to yellow. First, the antioxidant activity of antioxidant vesicles alone was evaluated at different concentrations, and then in the presence of an amount of CUR equal to that entrapped in the vesicle bilayer. 

The antioxidant activity of empty and un-functionalized niosomes was also studied and the obtained results confirmed that the vesicle based on the Tween 80 surfactant did not show intrinsic scavenging activity (data not shown).

**Figure 3:**
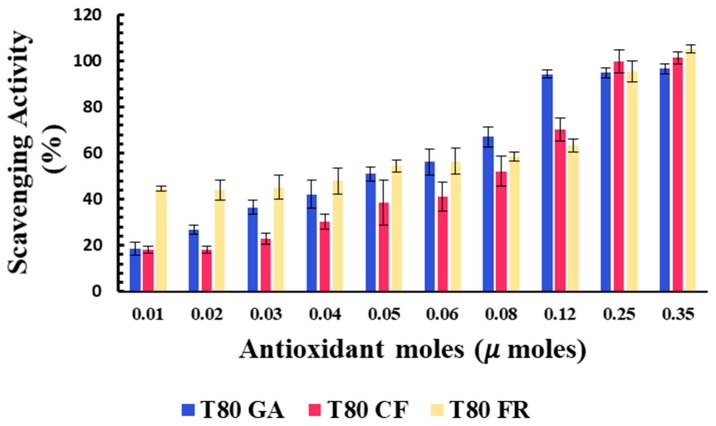
Antioxidant activity of T80-GA (blue), T80-CF (red), and T80-FR (yellow) against DPPH• expressed as a percentage of radical scavenging activity versus antioxidant µmoles grafted onto niosomes’ surfaces. Results represent the mean ± S.D. of three independent experiments performed with triplicate measurements.

The percentages of the DPPH scavenging activity of niosomes in the functionalization of antioxidant moles grafted onto the vesicle surfaces are reported in Figure 3. All niosomal formulations showed a dose-dependent DPPH scavenging activity.

Data reported in Figure 3 show that the covalent grafting of phenolic acids on a niosome’s surface represent a successful strategy to develop nanomaterials with intrinsic antioxidant properties. To the best of our knowledge, this study provides the first proof of concept of niosomes with intrinsic free radical scavenging properties that could be used as versatile platforms for pharmaceutical, cosmetic, and nutraceutical applications.

The antioxidant activity of the designed formulation was compared considering the 50% of inhibition of DPPH radicals in the functionalization of the moles of antioxidants grafted onto niosomes’ surfaces (SC50).

Better antioxidant activity was observed for T80-FR and T80-GA, as indicated by their SC50 values equating to 0.041 and 0.051 µmoles, respectively (Table 3). On the contrary, the DPPH scavenging ability of T80-CF was lower, in respect to the other formulations: in fact, the higher value of SC50 equating to 0.087 µmoles highlighted a reduced ability to scavenge free radicals. For the vesicles functionalized with 0.03 µmoles of phenolic acids on their surfaces, the scavenging activity against DPPH radicals, indeed, was 45.18% and 36.47%, respectively, for T80-FR and T80-GA, while for T80-CF, it was only approximately 22.83%.

**Figure 4:**
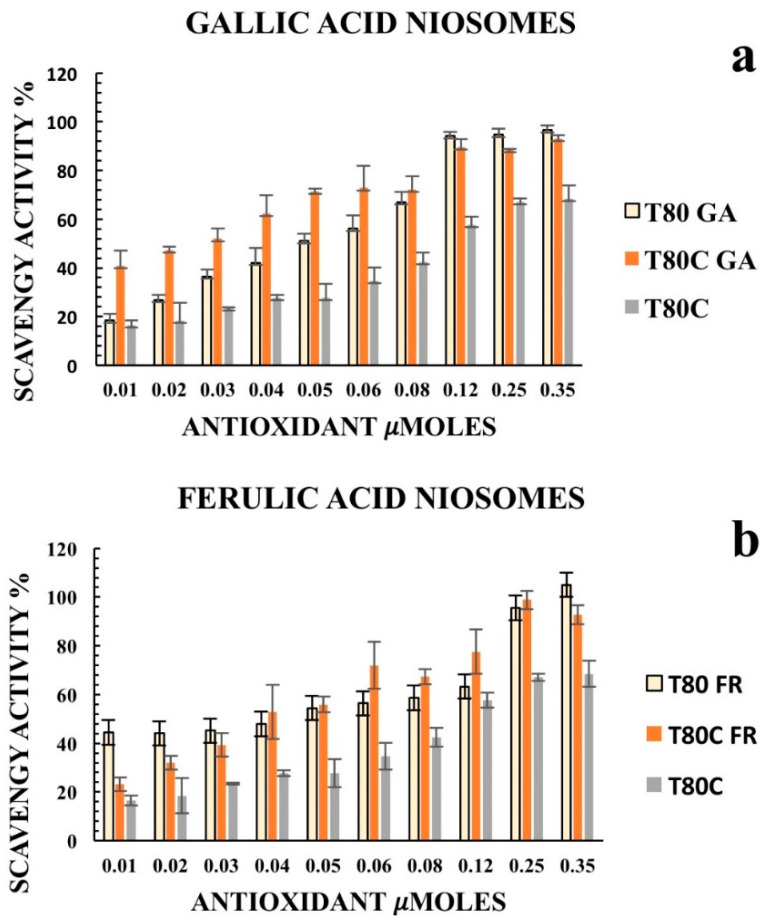

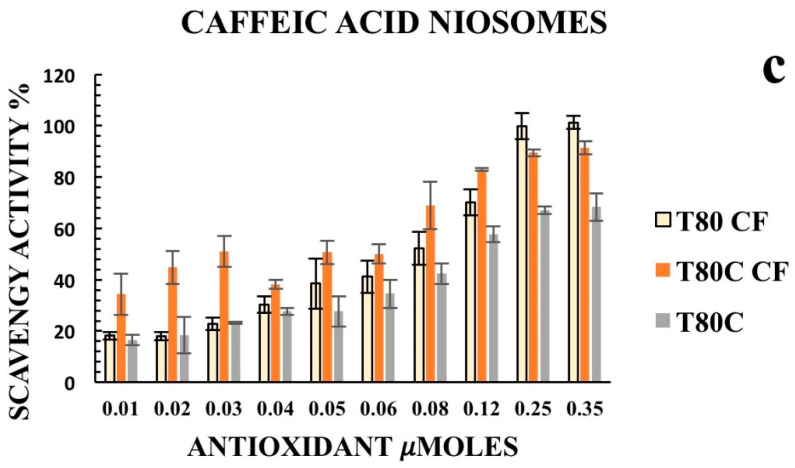
Antioxidant activity of empty (yellow) and CUR-loaded (orange) T80-GA (**a**), T80-FR (**b**), and T80-CF(**c**) against DPPH• expressed as a percentage of radical scavenging activity versus antioxidant µmoles grafted on the niosomes’ surfaces. Data represent the mean ± S.D. of three independent experiments performed with triplicate measurements.

Furthermore, we decided to enhance the antioxidant activity of the designed vesicles through the combination with CUR in order to exploit any potential synergic effect. The loading of CUR in the vesicle bilayers led to different responses, depending on the phenolic acids conjugated on the vesicles’ surfaces. In particular, data obtained for T80C-FR niosomes showed that CUR loading in the niosomal bilayer led to a reduced response. At the lowest concentration tested, indeed, the DPPH scavenging activity for T80C-FR was 23.16% against the 44.49% obtained for T80-FR. This trend could be due to a potential antagonistic effect of FR and CUR. On the contrary, a synergic effect for T80C-GA and T80C-CF was observed. In fact, the antioxidant activity of T80C-GA and T80C-CF was higher in respect to the sum of the effects of the singular components (Figure 4). For instance, at the lowest concentration tested, the scavenger ability of T80C-GA was 44.89%, against the percentage obtained with T80-GA and T80C, equating to 18.46% and 16.44%, respectively. In fact, samples loaded with CUR and externally grafted with CF and GA showed SC50 values that were statistically different (*p* < 0.05) in respect to the formulations loaded only with one antioxidant component (Table 3). The reduction in SC50 values suggested a better antioxidant activity when phenolic acid-grafted niosomes were used in combination with CUR, compared to the corresponding single-loaded formulations.

According to the results, CUR increased the antioxidant activity of T80-GA and T80-CF vesicles; this synergic effect allows its use to lower the amount of active substance required, while maintaining its antioxidant efficacy in clinical practice.

**Figure 5:**
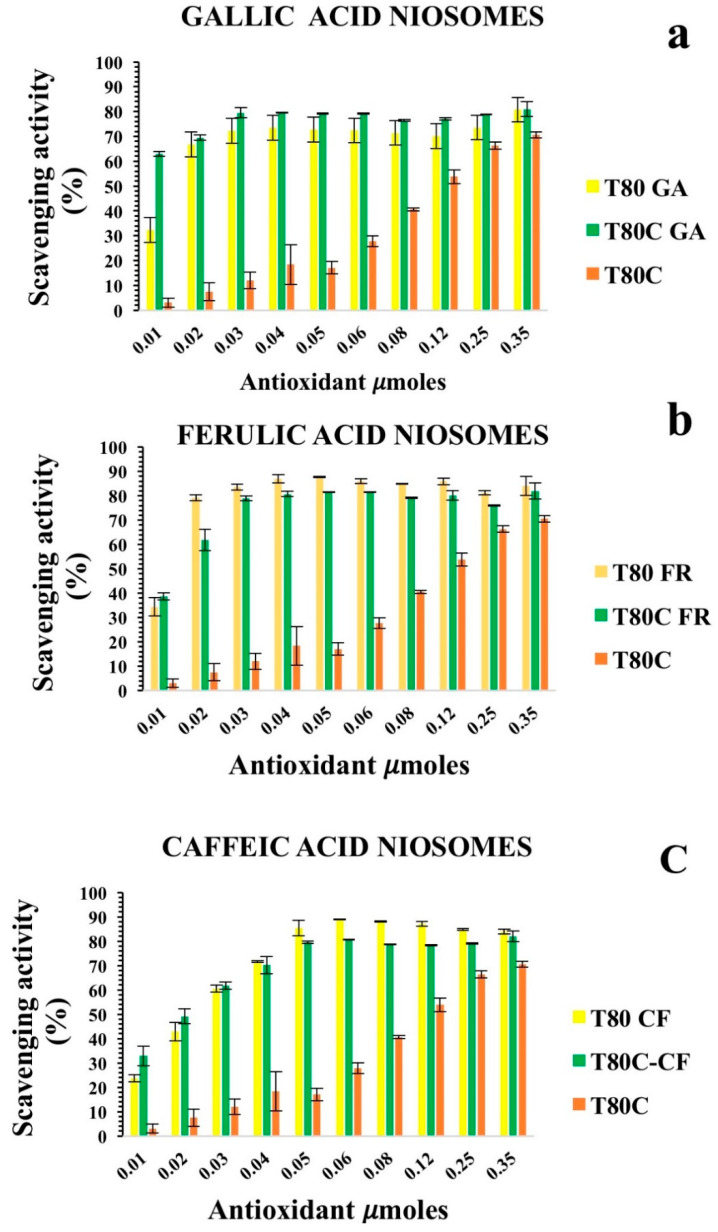
Antioxidant activity of empty (yellow) and CUR-loaded (orange) T80-GA (**a**), T80-FR (**b**), and T80-CF; (**c**) against ABTS• expressed as percentage of radical scavenging activity versus antioxidant µmoles grafted onto niosomes’ surfaces. Results represent the mean ± S.D. of three independent experiments performed with triplicate measurements.

Considering the multiple mechanisms involved in the antioxidant effect, a single test is not enough to evaluate the antioxidant capacities of the new materials. Consequently, we also performed an ABTS assay to obtain deeper insights. Polyphenolic acids retained their ABTS radical scavenging activity after conjugation on the niosomes’ surfaces (Figure 5). The results obtained with the ABTS assay were quite different compared to the DPPH assay since the formulations were more sensitive to the test. A higher antioxidant activity was recorded at the lowest concentration of antioxidants grafted onto the vesicles’ surfaces. In fact, SC50 values against ABTS free radicals were significantly lower in respect to the SC50 values against the DPPH radicals, indicating a better scavenging ability.

Both T80-GA and T80-FR with an SC50 of 0.014 µmoles exhibited better antioxidant ABTS radical scavenging activity than T80-CF (Table 3). These results were consistent with the DPPH assay, and also confirmed the synergic effect of CUR and GA: in fact, as indicated by the SC50 values reported in Table 3, T80C-GA was found to have an SC50 of 0.0057 µmoles, whereas T80-GA showed an SC50 of 0.014 µmoles and these data were statistically different. This synergic effect allows the use of lower amounts of antioxidants to obtain an enhanced radical scavenging efficacy. These results highlighted the possibility to use CF or GA and CUR together to obtain devices with enhanced therapeutic potential in different oxidative stress-related diseases. Nonetheless, no synergic effect was observed when CUR was loaded onto T80-CF niosomes.

Finally, these results indicated that the conjugation of phenolic acids on niosome surfaces provides them with antioxidant activity and thus makes it possible to use these devices for the treatment of pathologies related to free radicals. The obtained nanomaterials in which one phenolic moiety is exposed in the outer surface and a second antioxidant molecule is loaded onto the vesicle bilayer also showed better antioxidant activity in respect to the devices endowed with a single antioxidant component. This suggests the great potential of these nanodevices as promising tools for the treatment of diseases caused by oxidative stress through combined approaches.

### 3.3. In Vitro Release Studies

**Figure 6:**
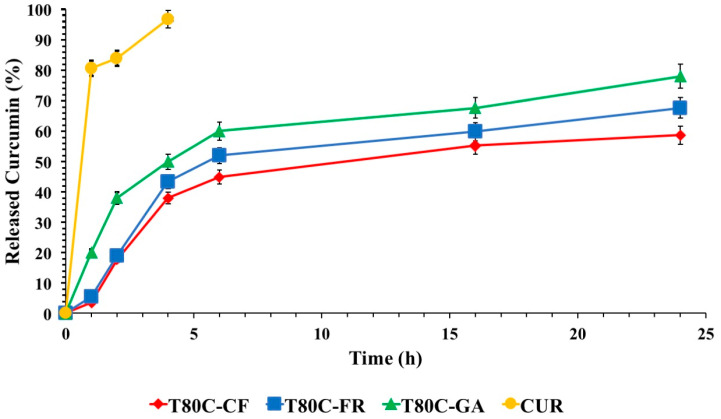
In vitro CUR release in phosphate buffer pH 7.4 (Tween 80 0.5%) at 37 °C from T80C-GA (▲), T80C-CF (♦), T80C-FR (■), CUR solution (•). In all cases, each value represents the mean ± S.D. of three independent experiments.

In vitro drug release profiles of CUR from vesicles are shown in Figure 6. The release of free CUR was used as a control and the total amount of drug was released in 4 h. As expected, CUR release from niosome samples was retarded, compared to that obtained by a drug-free solution. These results indicated that the designed vesicles can control and sustain the CUR release in time, suggesting the role of these devices as delayed delivery systems. FR- and CF-functionalized systems showed similar drug release trends. On the contrary, the release from T80C-GA was the highest, achieving 78% of the drug released after 24 h. These different patterns could be ascribed to the different lipophilicity of the phenolic acids conjugated on the vesicle surfaces. As previously mentioned, the hydrophobicity of molecules conjugated on the vesicle surfaces affected the size. Probably, the presence of GA, with a lower hydrophobic character, on the niosomal surface led to the formation of a layer with lower cohesion and, consequently, a higher drug diffusion rate in respect to the layer formed by FR and CF.

## 4. Conclusions

In this work, we prepared the first example of niosomes with intrinsic antioxidant activity by chemical conjugation of phenolic acids onto the vesicle surface.

The data obtained confirmed our hypothesis that surface functionalization of niosomes provides them with antioxidant activity and offers the possibility of using these devices for the treatment and prevention of diseases associated with oxidative stress. Moreover, these vesicles may be used as carriers of other biologically active molecules for multiple and synergic drug therapy. Curcumin, loaded in these formulations, was used as a model drug and the results highlighted an important synergic antioxidant effect.

## Figures and Tables

**Table 1:** Average size, polydispersity index (P.I), zeta potential, and grafting efficiency of empty niosomes before and after conjugation with phenolic acids at 25 °C. Data represent the mean ± S.D. of three independent experiments performed with triplicate measurements.

Formulations	Size (nm)	P.I.	Zeta Potential (mV)	Grafting Efficiency (%)
T80	549.8 ± 16.7	0.265 ± 0.010	−30.1 ± 0.577	-
T80-GA	495.8 ± 12.0	0.219 ± 0.016	−13.4 ± 0.2	36.1 ± 4.14
T80-CF	433.8 ± 16.6	0.187 ± 0.011	−11.1 ± 0.1	52.5 ± 0.03
T80-FR	373.1 ± 11.1	0.259 ± 0.013	−17.6 ± 0.5	44.9 ± 7.05

**Table 2:** Physicochemical properties (hydrodynamic diameter, polydispersity index (P.I.), and zeta potential) of curcumin-loaded niosomes before and after conjugation with phenolic acids at 25 °C. Data represent the mean ± S.D. of three independent experiments performed with triplicate measurements.

Formulations	Size (nm)	P.I.	Zeta Potential (mV)	Grafting Efficiency (%)
T80C	201.25 ± 9.32	0.251 ± 0.036	−27.8 ± 0.283	-
T80C-GA	91.70 ± 4.50	0.225 ± 0.042	−13.3 ± 1.04	14.6 ± 3.15
T80C-CF	83.70 ± 2.60	0.273 ± 0.007	−14.9 ± 0.850	50.8 ± 0.09
T80C-FR	72.5 ± 1.11	0.281 ± 0.018	−15.9 ± 0.404	41.4 ± 5.07

**Table 3:** SC50 values of antioxidant-functionalized niosomes in DPPH and ABTS scavenging assay. Results represent the mean ± S.D. of three independent experiments performed with triplicate measurements. * *p* < 0.05 phenolic acid-conjugated T80 niosomes vs. phenolic acid-conjugated T80C niosomes.

Sample	SC50 for DPPH (µmoles)	SC50 for ABTS (µmoles)
T80-FR	0.041 ± 0.004	0.014 ± 0.1
T80C-FR	0.041 ± 0.004	0.013 ± 0.1
T80-GA	0.051 ± 0.005 *	0.014 ± 0.1 *
T80C-GA	0.021 ± 0.002	0.0057 ± 0.0006
T80-CF	0.087 ± 0.009 *	0.025 ± 0.002
T80C-CF	0.047 ± 0.005	0.026 ± 0.003

## Data Availability

All data presented in this study are included within the article.

## Supplementary Materials

The following are available online at https://www.mdpi.com/article/10.3390/pharmaceutics13060766/s1.
