# Supplementary material for: New Nanomaterials with Intrinsic Antioxidant Activity by Surface Functionalization of Niosomes with Natural Phenolic Acids
Source: Pharmaceutics. 2021 May 21;13(6):766. doi: 10.3390/pharmaceutics13060766 (PMC8224007; doi:10.3390/pharmaceutics13060766)
Supplement: Supplementary file 1 [file pharmaceutics-13-00766-s001.zip › pharmaceutics-1223892-supplementary.pdf]

pharmaceutics

Supplementary Materials: New Nanomaterials with Intrinsic Antioxidant Activity by Surface Functionalization of Niosomes with Natural Phenolic Acids

Elisabetta Mazzotta, Carla Orlando and Rita Muzzalupo

Preparation of niosomes

Multilamellar niosomes vesicles (MLVs) were prepared using thin film hydration method and Tween80 as surfactant at 10 mM total lipid concentration. In order to obtain CUR-loaded niosomes, 2.7×10^{-6} moles of CUR were added to the surfactant methanol solution and, after vacuum evaporation, the film was hydrated with 10 mL of distilled water at 60 °C for 30 min to form large empty MLVs. Small unilamellar vesicles (SUVs) were obtained starting from MLVs by sonication in an ultrasonic bath at 60 °C for 30 min. After preparation, the dispersions were left to equilibrate at room temperature overnight to allow complete annealing and partitioning of the drug between the lipid bilayer and the aqueous phase. The purification of niosomes was carried out by a flow of niosomes across a Sepharose CL-4B gel column and after this, the formulations were stored in the dark at 4 °C until their use in subsequent experiments.

Entrapment Efficiency

CUR entrapment efficiency was reported as the percentage of drug loaded into the purified niosomes compared to the total amount of drug presented in the non-purified samples. Consequently, 0.1 mL of purified and non-purified sample were diluted in 10 mL of ethanol in order to allow the break of vesicle bilayer and the release of encapsulated CUR. The amount of drug in these solutions was then measured using UV-vis spectrophotomer at 426 nm. The experiment was run in triplicate and the results were expressed as mean ± SD.

Physicochemical Characterization

Hydrodynamic diameter of niosomes was determined by dynamic light scattering (DLS) using a 90 Plus Particle Size Analyzer (Brookhaven Instruments Corporation, New York, USA) while zeta potential was also measured using the Zeta-sizer Nano-ZS ZEN 3600 (Malvern Instruments Ltd., Malvern U.K.) after 100 times dilution by distilled water, at 25 ± 0.1°C. Data were reported as the mean of three independent experiment carried out in triplicate.

Determination of Total Phenolic Content

Total phenolic content was evaluated by Folin-Ciocolteau method. 0.5 mL of Folin-Ciocolteau reagent was added to 0.5 mL of the samples and left in the dark for 5 min. Then, 3 ml of Na_2CO_3 at 20% and 5.5 mL of distilled water were added and incubated in the dark for 20 min. The sample were centrifuged at 3500 rpm for 10 min and the absorbance of the supernatant was measured spectrophotometrically at 750 nm. A calibration curve using GA was carried out and the total phenolic content are expressed as GA equivalents (GAE).

DPPH Radical Scavenging Activity Assay

The antioxidant activity was determined by using DPPH assay according to the method reported by Tavano et. Al. Briefly, different stock solutions for each antioxidant-functionalized niosomes (containing different moles of antioxidant-functionalized vesicles) were prepared by dilution from the initial niosomal solution. 1.5 mL of the sample

were incubated with 1.5 mL of ethanol DPPH solution 0.25 mM at room temperature in the dark. After 30 min, absorbance measurements were taken at 517 nm with UV-vis spectrophotometer. The DPPH radical scavenging activity was calculated according to the following equation:

$$Scavenging\ activity\ (\%) = \frac{(A^{\circ} - A^{1})}{A^{\circ}} \times 100$$

where A° is the absorbance of control (blank, without niosomes) and A^1 is the absorbance in presence of niosomal formulations. Each experiment was carried out in triplicate and the results expresses as means ± SD

ABTS Radical Scavenging Assay

Antioxidant activity of developed nanocarriers was estimated measuring their capacity to scavenge free radical ABTS•. A stock solution of 7 mM ABTS was mixed with potassium persulfate 2.45 mM and stirred in the dark for 12 h in order to generate ABTS• free radical. Before the use, the solution was diluted with ethanol until an absorbance of 0.7. Then, 1 mL of the samples were added to 3 mL of the ABTS•+ solution and after 6 min the absorbance was evaluated at 734 nm using a UV spectrophotometer.

Finally, ABTS scavenging activity was calculated, using the equation:

$$\%\text{inhibition} = \left(\frac{A_0 - A_S}{A_0}\right) \times 100$$

Where, A_s is the absorbance of the sample at 734 nm and A_0 is the control. All tests were realised in triplicate and the results expressed as means ± SD

In Vitro CUR Release Studies

CUR release from antioxidant-vesicles was examined under sink condition. Aliquots of niosomal samples (0.4 mL) were placed in dialysis bags and suspended in 20 mL of phosphate buffer pH 7.4 containing 0.5 % of Tween80 under stirring at 37 °C. At specific time points, 2 mL of the medium were taken and withdrawn with fresh buffer. The amount of CUR in the withdrawn samples was analysed by using UV-vis spectrometer at 426 nm. Each experiment was done in triplicate and the results were reported as mean ± SD.
